# Regulation of Epithelial Differentiation in Rat Intestine by Intraluminal Delivery of an Adenoviral Vector or Silencing RNA Coding for Schlafen 3

**DOI:** 10.1371/journal.pone.0079745

**Published:** 2013-11-11

**Authors:** Pavlo L. Kovalenko, Lisi Yuan, Kelian Sun, Lyudmyla Kunovska, Sergey Seregin, Andrea Amalfitano, Marc D. Basson

**Affiliations:** 1 Department of Surgery, Michigan State University, East Lansing, Michigan, United States of America; 2 Research Service, John D. Dingell Veterans Affairs Medical Center, Detroit, Michigan, United States of America; 3 Department of Microbiology and Molecular Genetics, Michigan State University, East Lansing, Michigan, United States of America; University of Florida, United States of America

## Abstract

Although we stimulate enterocytic proliferation to ameliorate short gut syndrome or mucosal atrophy, less effort has been directed at enterocytic differentiation. Schlafen 3 (Slfn3) is a poorly understood protein induced during IEC-6 enterocytic differentiation. We hypothesized that exogenous manipulation of Slfn3 would regulate enterocytic differentiation *in vivo*. Adenoviral vector coding for Slfn3 cDNA (Ad-GFP-Slfn3) or silencing RNA for Slfn3 (siSlfn3) was introduced intraluminally into rat intestine. We assessed Slfn3, villin, sucrase-isomaltase (SI), Dpp4, and Glut2 by qRT-PCR, Western blot, and immunohistochemistry. We also studied Slfn3 and these differentiation markers in atrophic defunctionalized jejunal mucosa and the crypt-villus axis of normal jejunum. Ad-GFP-Slfn3 but not Ad-GFP increased Slfn3, villin and Dpp4 expression in human Caco-2 intestinal epithelial cells. Injecting Ad-GFP-Slfn3 into rat jejunum *in vivo* increased mucosal Slfn3 mRNA three days later vs. intraluminal Ad-GFP. This Slfn3 overexpression was associated with increases in all four differentiation markers. Injecting siSlfn3 into rat jejunum *in vivo* substantially reduced Slfn3 and all four intestinal mucosal differentiation markers three days later, as well as Dpp4 specific activity. Endogenous Slfn3 was reduced in atrophic mucosa from a blind-end Roux-en-Y anastomosis in parallel with differentiation marker expression together with AKT and p38 signaling. Slfn3 was more highly expressed in the villi than the crypts, paralleling Glut2, SI and Dpp4. Slfn3 is a key intracellular regulator of rat enterocytic differentiation. Understanding how Slfn3 works may identify targets to promote enterocytic differentiation and maintain mucosal function *in vivo*, facilitating enteral nutrition and improving survival in patients with mucosal atrophy or short gut syndrome.

## Introduction

The intestinal lumen is lined by a specialized simple columnar epithelium that performs the primary functions of digestion and water and nutrient absorption and forms a barrier against luminal pathogens [1]. Critically ill patients require total parenteral nutrition (TPN) until bowel function returns [2]. Prolonged TPN may be required for patients with short gut syndrome awaiting sufficient intestinal adaptation for completely enteral nutrition [3], [4]. This can take up to 3 years, and less fortunate patients will require permanent TPN, with risks of complications and very high cost [5]. Growth hormone may enhance adaptation by promoting enterocytic proliferation, increasing the number of enterocytes available for nutrient absorption and digestion, but its clinical efficacy seems limited [4]. The Glucagon-like peptide-2 (GLP-2) analog teduglutide also stimulates enterocytic proliferation [6]. An alternative or complementary approach would be to promote enterocytic differentiation, increasing the potency of individual enterocytes, but little is known about how to promote enterocytic differentiation in vivo.

Slfn3 belongs to a superfamily of growth regulatory genes first discovered in mice [7]. Their function is poorly understood. A unique domain named “Schlafen box” along with the adjacent ATP/GTP binding AAA domain is common to all members of the family [8]. The Schlafen superfamily is divided into three subgroups based on overall exon homology and size of the encoded proteins [7], [8]. Slfn3, along with Slfn4, Slfn6, and Slfn7, belongs to the intermediate subgroup. Slfn3 has recently been associated with decreased cancer stem cell marker expression in FOLFOX-resistant colon cancer cells [9]. Other Schlafens such as Slfn1 or Slfn8 may modulate T cell development [7], [10]. Slfn2 expression has been reported to increase during cellular differentiation in haematopoietic cell lines [11], and Slfn2 induction may contribute to the differentiation of monocytes and macrophages to osteoclasts induced by receptor activator of NF- *𝛋*B ligand (RANKL) [12].

We previously reported that diverse stimuli of intestinal epithelial differentiation (butyrate, transforming growth factor beta (TGF-β), and cyclic strain) require Slfn3 induction to stimulate IEC-6 cells in vitro to overexpress villin and increase Dpp4 specific activity. An *in vivo* role for Slfn3 has not previously been described. We therefore sought to evaluate whether exogenous modulation of Slfn3 by direct intraluminal administration of an Ad vector coding for Slfn3 (Ad-GFP-Slfn3) or silencing RNA (siRNA) would change enterocytic differentiation *in vivo.* We studied villin, dipeptidyl-peptidase 4 (Dpp4), SI and glucose transporter 2 (Glut2) expression as markers of intestinal epithelial differentiation in vivo, and also evaluated intestinal epithelial morphology, proliferation, and aptoptosis. We further investigated changes in endogenous Slfn3 in a model of disuse mucosal atrophy, and also evaluated levels of AKT and p38 signaling in the atrophic mucosa since AKT and p38 have previously been implicated in the induction of Slfn3 by cyclic strain [13]. Understanding Slfn3-induced differentiation may allow us to manipulate enterocytic differentiation and improve mucosal function in vivo, facilitating enteral nutrition and improving survival in patients with mucosal atrophy or short gut syndrome.

## Methods

### Ethical Approval

All animal procedures were reviewed and approved by the Michigan State University Institutional Animal Care and Use Committee (AUF 02/13-018-00). Animal care was in accordance with standards of the Public Health Service and Association for Assessment and Accreditation of Laboratory Animal Care International.

### Adenovirus vector construction

The Slfn3 cDNA of the rat Slfn3 gene was subcloned in-frame into pAdTrackCMV shuttle plasmid, linearized and recombined into the Ad genome containing pAdEasy1 to create Ad-GFP-Slfn3. Ad vector production, purification and characterization was performed as described previously [14].

### Cell culture

Caco-2 intestinal epithelial cells (CRL-2102, American type Culture Collection, Manassas, VA) a common model of intestinal epithelial biology able to differentiate in culture [15] were maintained at 37°C 5% CO_2_ as previously described [16]. 80% confluent Caco2 cells were treated with 4000 virus particles of Ad-GFP (Control) per cell or Ad-GFP- Slfn3 for 48 hours.

### Animal procedures

8–14 weeks-old male Sprague Dawley rats were used for the experiments. Animals were deprived of food overnight before surgery. All operative procedures were performed under isoflurane anesthesia, and all efforts were made to minimize suffering. For viral-induced expression experiments, 10^11^ vector particles (5.0× 10^12^ particles/kg) expressing Ad-GFP-Slfn3 or Ad-GFP (control) in 200 µL of PBS were injected intraluminally into temporarily obstructed jejunal segments of anesthetized rats at laparotomy (n = 7 per group). Similarly, for Slfn3 silencing experiments 100 nM of Slfn3 siRNA or Non Targeting-1 (NT1) siRNA (Dharmacon, Lafayette, CO) in 200 µL, prepared using Oligofectamine according to the manufacturer's protocol, were injected and confined for 30 minutes in the 3 cm long region of jejunum of anesthetized rats at laparotomy (n = 6–10). Postoperatively, the animals were given water and chow diet (Research Diets, New Brunswick, NJ) ad libidum. Control and experimental rats were sacrificed 72 hours after injection. Animals were fasted overnight before harvest.

To study Slfn3 changes during mucosal atrophy, we created defunctionalized segments of jejunum in continuity with the remainder of the bowel as previously described [17]. Laparotomy was performed in isoflurane-anesthetized rats. The proximal jejunum was anastomosed to the distal jejunum seven cm more distally than the original transection with a side to side anastomosis using running 7–0 vicryl sutures. Postoperatively, the animals were given water and liquid diet (Jevity 1, Abbot Nutrition, Columbus, OH) ad libidum. Control and experimental rats were sacrificed 72 hours after injection. Animals were fasted overnight before harvest. Mucosal scrapings and tissues were collected and processed for RT-PCR, Western blots, or immunohistochemistry at the indicated time points.

### Dpp4 specific activity assay

For Dpp4 activity studies 100 nm of NT1 or Slfn3 siRNA in 200 µL, prepared using Oligofectamine according to the manufacturer's protocol, were injected into temporarily obstructed segments of jejunum, proximal ileum, mediate ileum, lower ileum or colon of anesthetized rats at laparotomy for 30 minutes. Control and experimental rats were sacrificed 48 hours after injection. Dpp4 activity was measured using the Dpp4-Glo assay according to the manufacturer's protocol (Promega, Madison, WI). Standard Dpp4 enzyme was purchased from Sigma. 10 mg of tissue were harvested in 0.5 ml of ice cold PBS and homogenized using a Bulletblender (Next Advance, Averill Park, NY). Dpp4 activity was measured in 50 µl of centrifuged sample diluted 30× in PBS.

### Western Blot Analysis

Mucosal scrapings from target intestinal segments after harvest were immediately immersed in ice cold lysis buffer [17]. Tissue was homogenized using a BulletBlender (Next Advance, Averill Park, NY) and then centrifuged at 15,000 g for 10 min at 4°C. Cultured Caco-2 cells were lysed in lysis buffer, centrifuged at 15,000 g for 10 minutes at 4°C, resolved by SDS-PAGE and transferred to Hybond ECL nitrocellulose membrane (Amersham Pharmacia Biotech, Piscataway, NJ) as previously described [17]. Nonspecific binding sites were blocked for 1 h at room temperature using Odyssey Blocking Buffer (Licor, Lincoln, NE). Membranes were probed with antibodies to Slfn3, SI, Glut2, Dpp4, villin (Santa Cruz Biotechnology, Santa Cruz, CA), phosphorylated AKT (pAKT, Thr308), total AKT, phosphorylated p38(p–p38, Thr180/Tyr182), total p38, phosphorylated FAK (pFAK, Tyr576) and total FAK as well as appropriate secondary antibodies. Bands were visualized using the Odyssey imaging system (Licor, Lincoln, NE) and analyzed with the Kodak Image Station 440CF.

### RNA isolation and qRT-PCR

Total RNA was isolated from the cells and tissues using Tri-Reagent (Molecular Research Center, Inc., Cincinnati, OH) in accordance with the manufacturer's instructions. Laser capture microdissection (LCM) was used to separate villous and crypt compartments from the rat jejunal mucosa. RNA from frozen sections LCM was isolated using CapSure HS LCM Caps (Arcturus, Mountain View, CA) and purified using the RNeasy Micro Kit (Qiagen, Valencia, CA). cDNA was prepared from RNA samples as we have described previously [18]. cDNA samples were analyzed by RT-PCR analysis using the BioRad MyiQ Real-Time PCR system and the BioRad SYBR Green supermix (BioRad Laboratories, Hercules, CA). Expression levels were determined from the threshold cycle (Ct) values using the method of 2^−ΔΔCt^ [18] using 18S expression as the reference control gene. Primer information as a follow: rat Slfn3 primers used were 5′-ATTCTGCTGTGCAGTGTTCG-3′ (upstream) and 5′-TTGCTTGGAGAAACATGCTG-3 (downstream). The ribosomal protein S18 (18S) 5′-CCCAGCACAATGAAGATCAA-3′ (upstream) and 5′-ACATCTGCTGGAAGGTGGAC-3′ (downstream).

Glut2 forward 5′-TTAGCAACTGGGTCTGCAAT-3′, reverse 5′-GGTGTAGTCCTACACTCATG-3′; Villin, forward 5′-CAACTTCTATGAGGGAGACTGGTAC-3′, reverse 5′-TAGTGATCCAGCTGTGTGGTATAGA-3′; SI, forward – 5′-GCAGAAGGCTACATGGA-3′, reverse – 5′-CCTTGCGACTGTCTCA-3′, Dpp4, forward – 5′-AGTTCTCGCGCTATCAGCGGC-3′, reverse – 5′-TCTCCGCGCGCGTGACTTCT-3′. The cycle conditions for the PCR were 1 cycle of 3 minutes at 95°C and 40 cycles of 30 seconds at 95°C, 30 seconds at the annealing temperature (57°C), and 30 seconds at 72°C.

### Histology, Immunohistochemistry, Ki67, TUNEL staining and Image Analysis

Three days after exposure to the virus or siRNA, animals were sacrificed, and the treated intestinal segments of jejunum were excised, fixed in 10% formalin for 24 hours, and embedded in paraffin. 5 µm sections through the area of intraluminal injection were placed on slides, deparaffinized, rehydrated, and utilized for various assays as described previously [19]. Changes in Slfn3, SI, Glut2 and villin were evaluated by subjecting sections to immunohistochemistry with antibodies directed against Slfn3, SI, Glut2, villin (Santa Cruz Biotechnology, Santa Cruz, CA), used at a dilution of 1:50 in PBS followed by Vectastain Universal ABC kit detection (Vector Labs, CA, USA). A scale of 0 to 4, with 0 as the lowest amount and 4 the highest positive staining was created for each staining [19]. Immunostained sections of the intestine were graded as previously described [19] by two independent observers blinded to the origin of the tissues at the time of scoring who have the same classification probabilities. Five views of villi were scored on each slide (representing one animal) at 200× magnification. Scores were analyzed by Chi square test at p<0.05. Alterations in proliferation were detected utilizing a Ki67 kit (Zymed, CA, USA), and apoptosis detection was accomplished by TUNEL staining (Calbiochem, CA, USA). Sections were counterstained with hematoxylin for Ki67, or methyl green for TUNEL, coverslipped, visualized and photographed on a Nikon Microphot-FXA (Nikon, Tokyo, Japan). Proliferation and apoptosis indices were measured as previously described [16].

### Statistical Analysis

Values are group means ± SE of the nontransformed data. Prior to analysis, all data were checked to ensure they fit a normal distribution. Statistical analysis was performed using unpaired *t*-tests or ANOVA as appropriate. Differences between means were considered significant at p<0.05.

## Results

### Ad-GFP-Slfn3 transfection increased Slfn3, villin and Dpp4 mRNA level in Caco-2 cells

**Figure 1:**
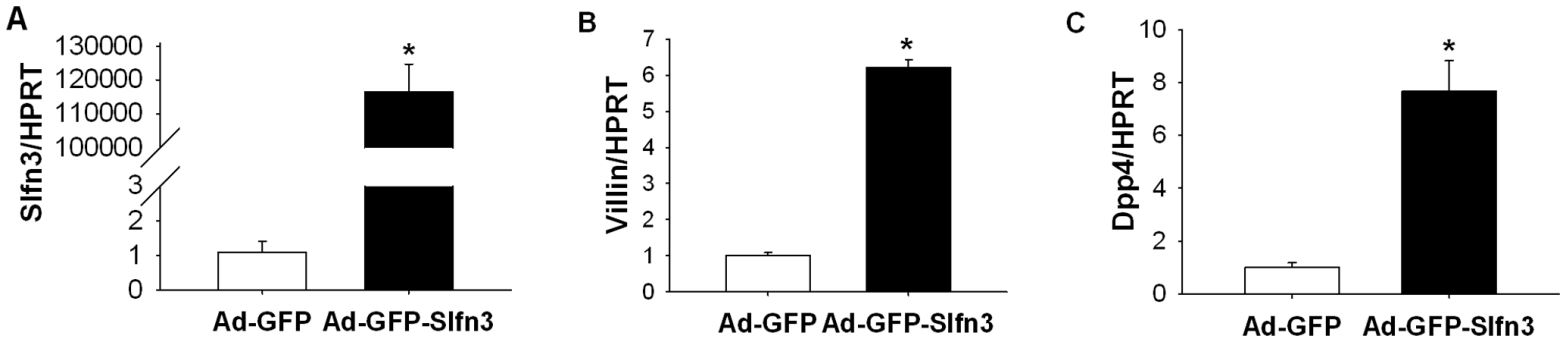
Slfn3 induction modulates Dpp4 activity in vitro. (A) 60–80% confluent monolayers of Caco-2 cells were infected with Ad-GFP-Slfn3 for 30 minutes. Ad-GFP-Slfn3 infection of Caco-2 cells increased Slfn3 gene expression compared to GFP-treated mock transfectants (n = 6, *p<0.05). Ad-GFP-Slfn3 infection of Caco-2 cells increased expression of villin (B) and Dpp4 (C) compared to GFP-treated control cells (n = 3, *p<0.01).

To first validate the function of the Ad-GPF-Slfn3 construct, we investigated whether infection of Ad-GFP-Slfn3 increases Slfn3 mRNA in Caco-2 intestinal epithelial cells. We subjected 60–80% confluent Caco-2 cells to 4000 vp/cell Ad-GFP-Slfn3 for 48 hours. Ad-GFP-Slfn3 infection of Caco-2 cells resulted in substantial measured Slfn3 transcript expression compared to Ad-GFP-treated transfectants (Fig. 1A, n = 6, p<0.05). Furthermore Ad-GFP-Slfn3 infection of Caco-2 cells also increased expression of the differentiation markers villin and Dpp4 compared to control (Fig. 1B-C, n = 3, p<0.01). Thus, we validated that exogenous overexpression of Slfn3 by direct infection of an Ad vector coding for Slfn3 cDNA would promote not only Slfn3 expression but also the phenotype of enterocytic differentiation in vitro in Caco-2 cells.

### *In vivo* delivery of Slfn3 by adenoviral vector increases the expression of differentiation markers in rat jejunal mucosa

**Figure 2:**
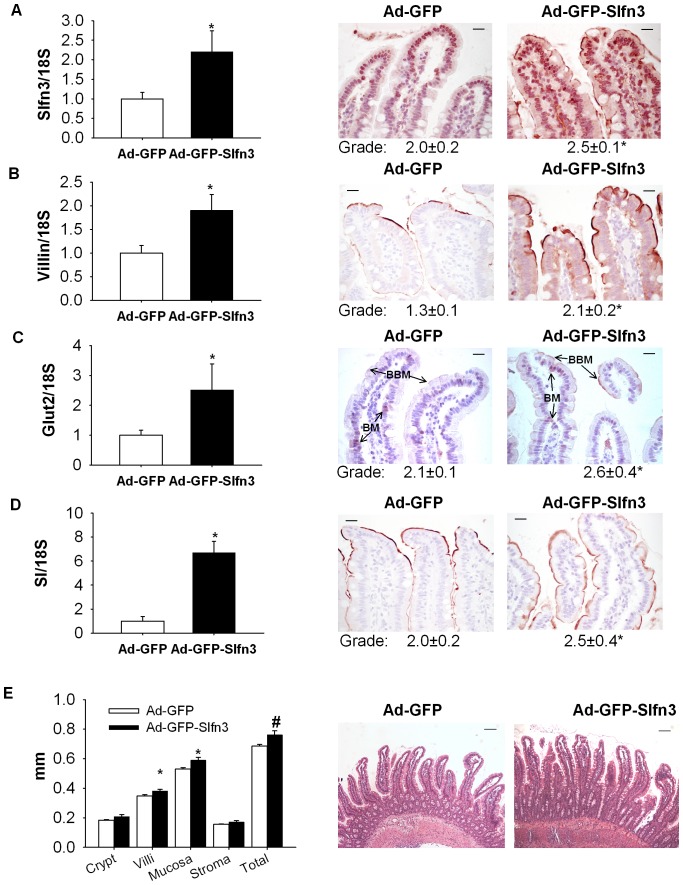
Slfn3 modulates expression of enterocyte differentiation markers in vivo. (A) 10^11^ vector particles in 200 µL of Ad-GFP or Ad-GFP-Slfn3 were injected intraluminally into temporarily obstructed jejunal segments of anesthetized 8-12 weeks old male rats. 72 hours after exposure to the Ad-GFP-Slfn3 virus there was a significant increase of Slfn3 transcript (A), villin (B), Glut2 (C), and SI (D) expression compared to respective mRNA from control animals inoculated with intraluminal Ad-GFP by real-time qPCR of mRNA from jejunal mucosal scrapings (n = 6–7, *p<0.01). Induced gene expression was confirmed by grading of slides stained with appropriate antibody. Overexpression of Slfn3 significantly increase expression of Glut2 (C) on brush border membrane (BMM, arrows) and compared to Ad-GFP treated mucosa. (F) Intestinal mucosa villous length was significantly increased after Ad-GFP-Slfn3 infection (n = 3, *p<0.05, #p<0.1). Representative images of control (Ad-GFP) and Ad-GFP-Slfn3 virus injected intestines. Scale bar in (A–E, 400× magnification) is 20 µm and in (F, 100× magnification) 100 µm.

Three days after exposure of the jejunal mucosa to the Ad-GFP-Slfn3 virus, we observed a 2.3±0.4 fold increase in Slfn3 transcript levels (Fig. 2A, n = 6–7, p<0.01). Ad-GFP-Slfn3 virus also induced a 1.8±0.4 fold increase in villin expression, a 3.8±0.3 fold increase in SI expression, a 2.9±0.6 fold increase in Glut2 expression compared to respective mRNA from control animals (Fig. 2B-D, n = 6–7, p<0.01 for each). We further investigated the effect of injecting the Slfn3-overexpressing virus on the protein level of these differentiation markers by IHC. Ad-GFP-Slfn3 injection significantly increased jejunal mucosal immunoreactivity for Slfn3, villin, Glut2, SI (p<0.05, n = 6–10 each). While in normal control rats GLUT2 was primarily located at the basement membrane (BM) overexpression of Slfn3 significantly increased availability of to the brush border membrane (BBM) (Figure 2C).

### Ad-GFP-Slfn3 infection causes minimal change in mucosal morphology and does not decrease proliferation or increase apoptosis

Morphometric analysis demonstrated slightly increased villus length (Fig. 2F, 0.38±0.01 mm after exposure to Ad-GFP-Slfn3 vs. 0.34±0.01 mm after Ad-GFP infection, (100× magnification, p<0.05). Length of the crypts did not change 3 days after Ad-GFP-Slfn3 infection (Fig. 2E).

To detect changes in proliferation 3 days after viral infection between Ad-GFP-Slfn3 injected rats and Ad-GFP injected rats, we immunostained sections from tissues with antibody against Ki67. Quantification demonstrated no decrease in Ki-67-positive intestinal mucosa epithelial cells in Ad-GFP-Slfn3-injected rats compared to control Ad-GFP-injected rats (data not shown). We next evaluated the possibility that intraluminal infection of Ad-GFP-Slfn3 in rat jejunum might influence apoptosis. TUNEL assay revealed no detectable increase in the number of apoptotic cells in rats exposed to Ad-GFP-Slfn3 compared to rats exposed to Ad-GFP (data not shown).

### Suppression of Slfn3 by siRNA in vivo reduced expression of differentiation markers

**Figure 3:**
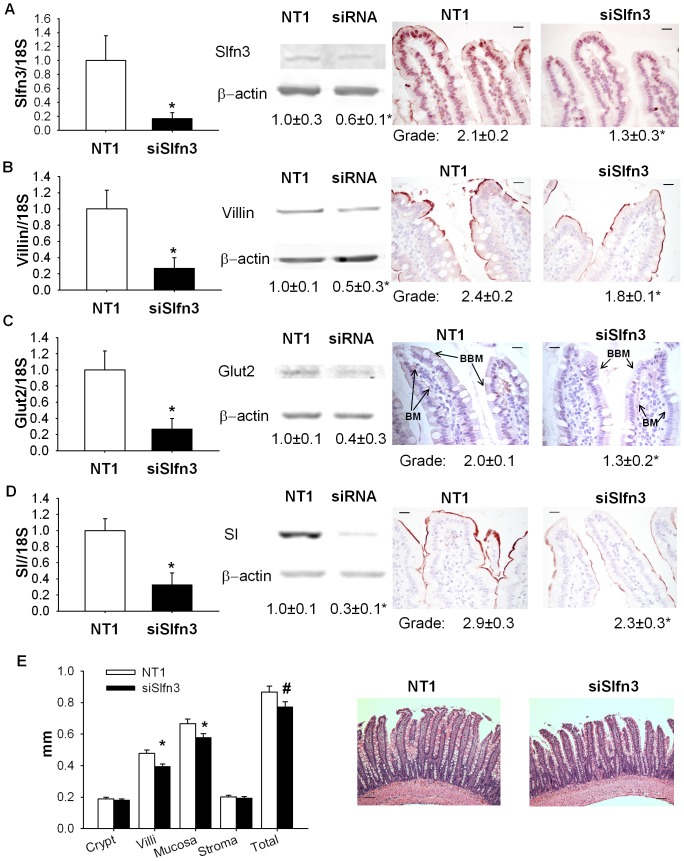
Suppression of Slfn3 reduces RNA and proteins level of Slfn3, Villin, SI and Glut2 in intestinal mucosa. (A) 100 nM of NT1 or Slfn3 siRNA (siSlfn3) were injected intraluminally into temporarily obstructed jejunal segments of anesthetized 12–14 weeks old male rats. 3 days after exposure of the jejunal mucosa to the siRNA, we observed substantial reduction of Slfn3 levels. By RT-PCR, Western blot and grading of stained slides, we observed increases in RNA and protein levels of villin (B), Glut2 (C), and SI (D) in mucosal homogenates (n = 6–10, *p<0.05). (E) Villus length and total mucosal thickness were reduced 3 days after injection of siRNA specific to Slfn3 (siSlfn3) (n = 9, *p<0.05, #p<0.1). Representative images of rat jejunum after injection of 100 nM of NT1 or siRNA for Slfn3. Scale bar in (A–D, 400× magnification) is 20 µm and in (E, 100× magnification) 100 µm.

Three days after transfection of the intestinal mucosa of rats with siRNA targeting Slfn3, Slfn3 mRNA levels were reduced 83.2±7.8% compared to the mucosa of rats exposed to NT1 siRNA (Figure 3A, n = 6-10, p<0.05). Transcript levels of other markers of differentiation were also reduced, as detected by RT-PCR. We observed a 71.6±10.9% reduction in villin transcript level, a 67.2±12.8% reduction in SI expression, and a 75±9.5% reduction of Glut2 expression in animals injected with intraluminal siRNA compared control rats inoculated with intraluminal NT1 (Fig. 3B-D, n = 6–7, p<0.01 for each). We obtained similar results using 18S, HPRT or RPLP0 transcript levels as controls for these gene expression studies. Only 18S-controlled results are shown here for brevity.

We also investigated the effect of Slfn3 inhibition on the protein level of Slfn3 and differentiation markers in rat intestine by Western blotting or immunohistochemistry with appropriate antibodies. 72 hours after exposure of the jejunal mucosa to nontargeting NT1 siRNA or siRNA targeting Slfn3, immunoreactivities for Slfn3, villin, and SI were all significantly reduced in the villi exposed to siRNA to Slfn3 (n = 7–13, Figure 3A–D, p<0.05) as detected by IHC. Suppression of Slfn3 significantly reduced expression of GLUT2 on the brush border membrane (BBM) and basal membrane (BM) of intestinal mucosa (Figure 3C).

Western blot analysis of the siRNA treated mucosa also revealed reduced protein levels of these differentiation markers compared to NT1 treated mucosa (n = 5, Figure 3A–D).

### Suppression of Slfn3 changes histology of the intestine

We also observed some morphological changes three days after transfection with siRNA. The length of the villi of the intestinal mucosa transfected with Slfn3 was 12.2±3.2% shorter than to the NT1 transfected controls (Fig 3E, p<0.05). Crypt depths in the intestinal mucosa did not differ after injection of siRNA to Slfn3.

### Slfn3 modulates level and activity of Dpp4 in segments of small intestine

**Figure 4:**
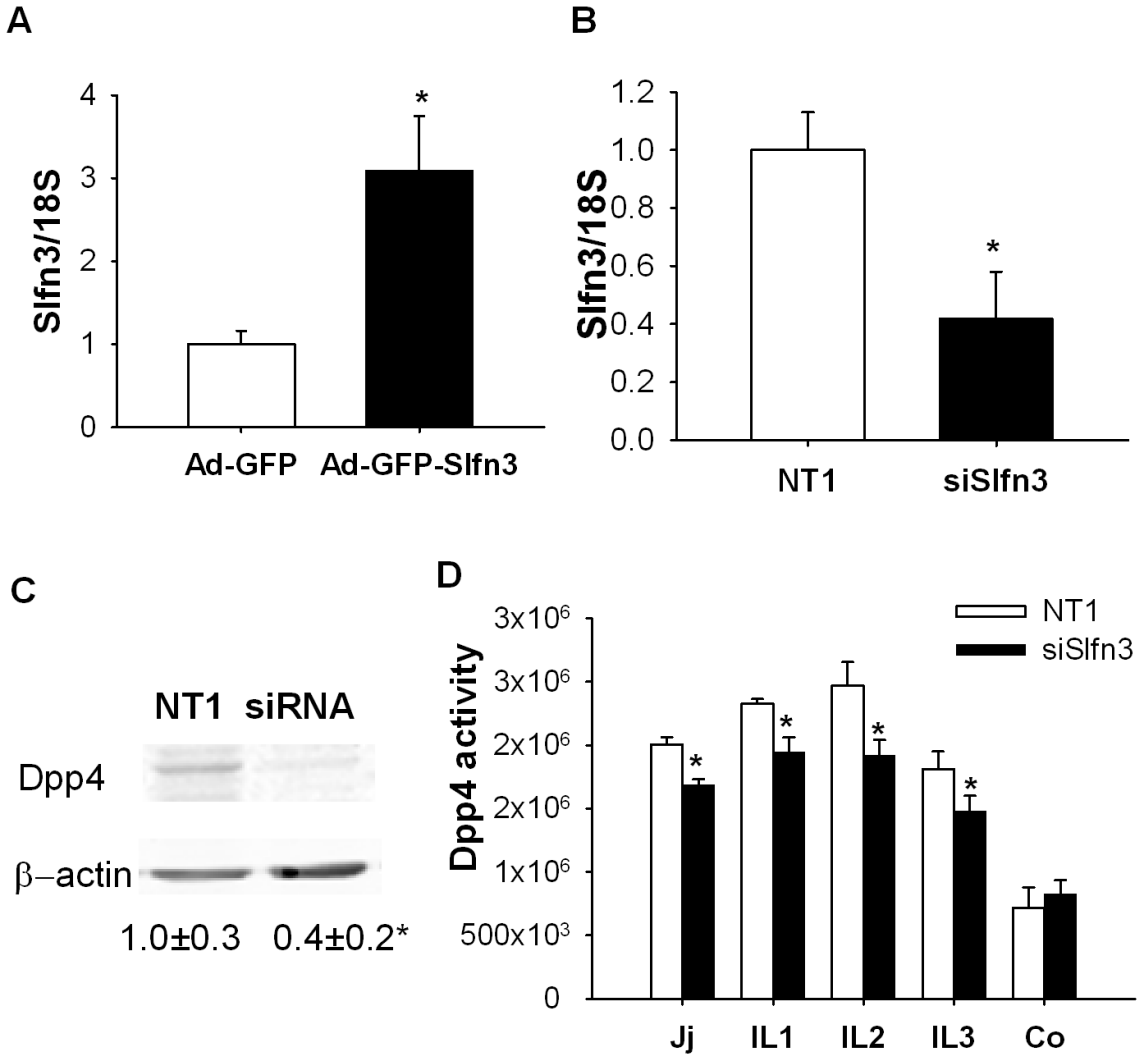
Effect of Slfn3 silencing on Dpp4 expression and activity. Exposure of the mucosa to Ad-GFP-Slfn3 increased transcript level of Dpp4 (A, n = 6, p<0.05).Silencing of Slfn3 reduced Dpp4 transcript (B) and protein levels (C n = 5–7, p<0.05). (D) Activity of Dpp4 was reduced 48 hours after injection of siRNA for Slfn3 in jejunum and ileum of rats, but not in the rat colon (n = 5, p<0.05).

Dpp4 is a brush border enzyme that cleaves NH_2_-terminal peptides from polypeptides. Three days after treatment of the jejunal mucosa with the Ad-GFP-Slfn3 virus, we observed a 4.0±1.0 fold increase in Dpp4 transcript levels (Fig. 4A, n = 6, p<0.05). We measured the effect of Slfn3 suppression on Dpp4 activity. Silencing of Slfn3 significantly reduced the RNA and protein levels of Dpp4 in the intestinal mucosa (Fig 4B-C, n = 6, p<0.05). The specific activity of the Dpp4 enzyme was also significantly reduced in mucosa from jejunal or ileal segments injected with siRNA to Slfn3 compared to NT1-injected intestinal mucosa (Fig 4D, p<0.05, n = 5). Interestingly, the injection of siRNA to Slfn3 did not alter Dpp4 specific activity in the colon.

### Endogenous Slfn3 is reduced in small intestinal mucosal atrophy

**Figure 5:**
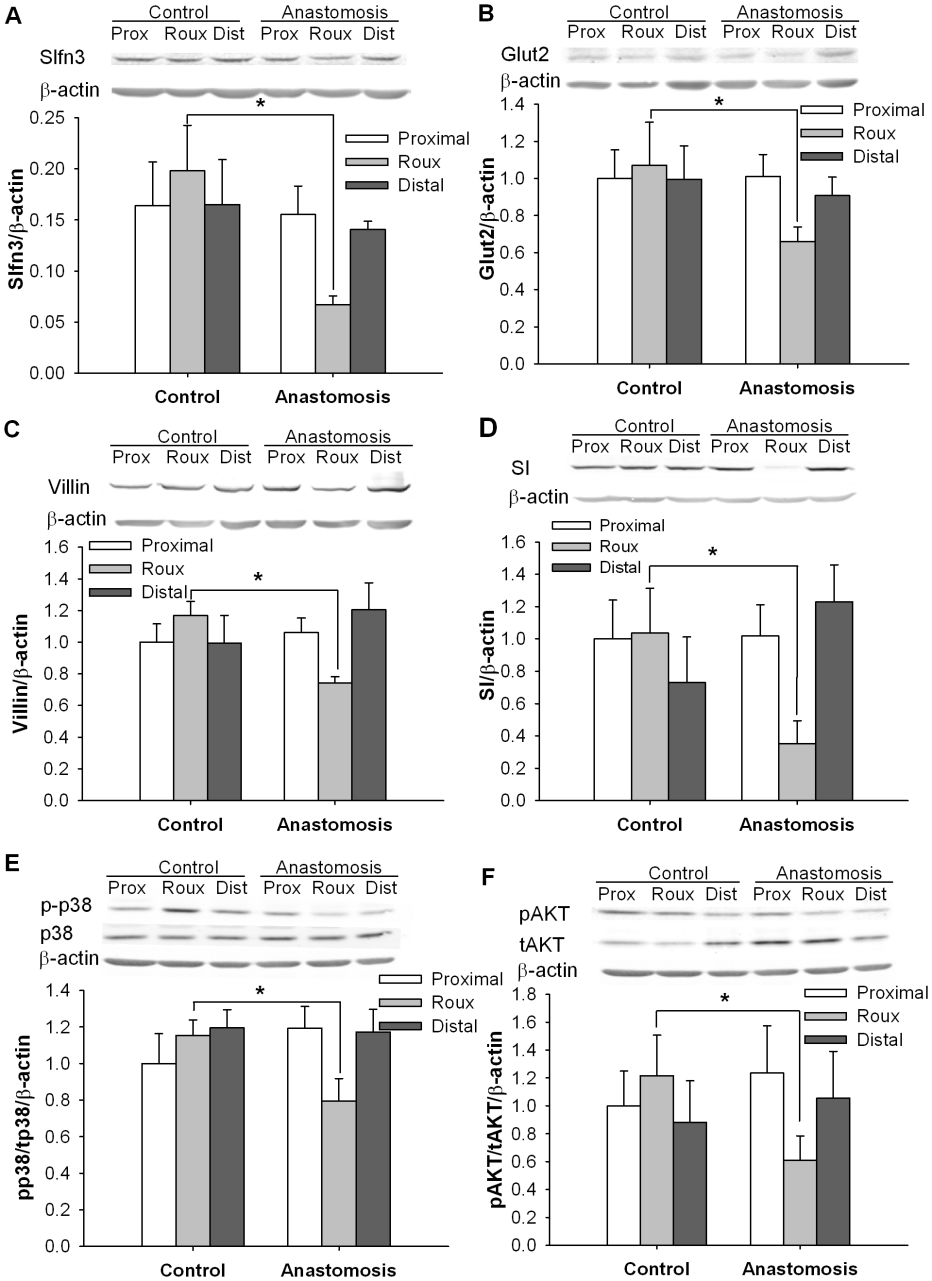
Changes of Slfn3, AKT and p38 signaling in defunctionalized intestine. Levels of Slfn3 (A), Glut2 (B), villin (C), SI (D), phosphorylated p38/total p38 (pp38/p38) (E), phosphorylated AKT/total AKT (pAKT/tAKT) (F) were lower in the intestinal mucosa of defunctionalized (Roux) bowel seven days after anastomosis compared to sham-operated controls or proximal or distal bowel in the same animals (p<0.05, n = 6). * - significantly different from area representing defunctionalized region of intestine in control (sham-operated) animals (p<0.05).

In further studies, we investigated the correlation between alterations in Slfn3 and differentiation marker expression in endogenous biology, examining both mucosal atrophy and differences between the intestinal crypts and villi. We induced mucosal atrophy in defunctionalized intestinal segments as previously described [17]. Seven days after defunctionalizing surgery, Slfn3 protein was 110±15.2% lower in the defunctionalized gut than in the same segment of bowel from sham-operated (control) animals. (Fig5A, n = 6, p<0.05). Seven days after anastomosis we also observed significant reduction of the Glut2, villin and SI differentiation markers compared to a similar area of the sham operated animals (Fig 5B–D, n = 4–8, p<0.05).

Since p38, AKT and FAK signaling have previously been implicated in the induction of Slfn3 in rat IEC-6 cells by repetitive deformation [13], we further investigated changes in these signals during mucosal atrophy. Indeed, levels of phosphorylated p38 relative to total p38 and of pAKT relative to total AKT were 21.5±9.6% and 45.3±8.7% respectively lower in the defunctionalized intestinal mucosa compared to the same segment of bowel from sham-operated animals in which Slfn3 was not reduced (n = 6, p<0.05, Fig 5B–C). In contrast, the level of pFAK-Y576 relative to total FAK was not significantly different between the defunctionalized and sham-operated mucosa (data not shown).

### Slfn3 levels correlate with expression of mucosal differentiation markers along the crypt-villus axis

**Figure 6:**
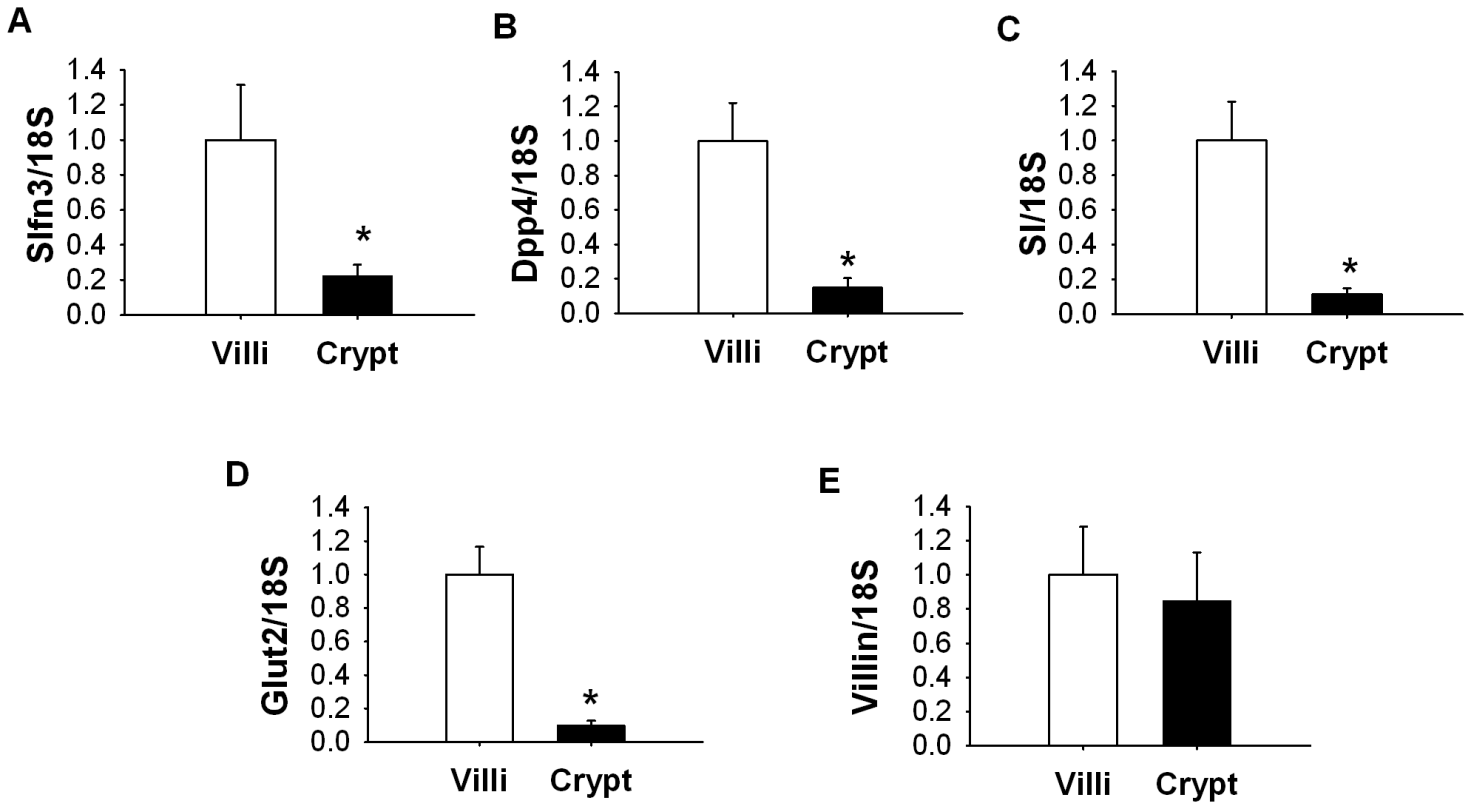
Difference in differentiation markers expression within intestinal mucosa. The level of Slfn3 was significantly lower in crypts of mucosa compared to villi (p<0.05, n = 6). The transcript levels of Dpp4 (B), SI (C), Glut2 (D) but not villin (E) were significantly lower in jejunal crypts compared to villi (p<0.05, n = 6).

In further studies, we used LCM to compare the expression of Slfn3 and of some enterocytic differentiation markers between the intestinal epithelial cells of the intestinal crypts and villi in normal rat jejunum. Slfn3 mRNA was 5.2±0.9 fold higher in the villi of the jejunal mucosa than in the crypts (Fig 6A, p<0.05, n = 6). Similarly expression of Dpp4 (Fig 6B), SI (Fig 6C) and Glut2 (Fig 6D) were 5.4±0.7, 6.7±0.8 and 9.1±0.5 fold higher respectively in the villi compared to the crypts. However no difference between crypts and villi was observed in the transcript level of villin (Fig 6E).

## Discussion

Although diverse hormones and growth factors have been used in attempts to overcome TPN-induced mucosal atrophy and augment adaptation, outcomes have been imperfect [20], [21], [22]. Current medical interventions mainly target proliferation, not differentiation, incompletely addressing the problem and conceivably risking eventual malignant transformation from mitogenic overdrive. Our results suggest that intramucosal modulation of Slfn3 by adenoviral delivery of Slfn3 or siRNA reduction of Slfn3 changes expression of several markers of intestinal epithelial differentiation in rat jejunal mucosa *in vivo.* In contrast, we observed only a modest lengthening of the villi and no effect on cell proliferation or apoptosis, suggesting that Slfn3 more specifically targets differentiation, although it is possible that more prolonged Slfn3 overexpression might alter mucosal proliferation and morphology as well.

The differentiated enterocyte exhibits a complex phenotype, and we cannot establish that Slfn3 drives every marker of such differentiation. However, brush border digestive enzymes such as Dpp4 and SI are canonical markers for enterocytic differentiation [23], [24], [25], [26], [27]. Dpp4 cleaves NH2-terminal dipeptides from polypeptides with either L-proline or L-alanine at the penultimate position, leading to their inactivation and/or degradation [28]. Dpp4 specific enzyme activity is also used in many other cell types as a marker of differentiation [29], [30]. For instance, loss or alteration of Dpp4 expression is linked to the development of cancers, including breast, prostate, lung, ovarian and hepatocellular cancer and melanoma, and plays a key role in tumorigenesis and metastasis [29]. Brush border hydrolases like SI and ion or carbohydrate transporters like Glut2 [31], [32] are also important membrane proteins that serve as common markers of intestinal epithelial differentiation and functional maturation.

In addition to brush border digestive enzyme activity, intestinal differentiation is also frequently assessed by villin expression [33], [34], [35], [36]. Villin is a key Ca^2+−^regulated actin binding protein in the microvillus core of the brush border [33], [34], [35], [36], [37], [38], [39], [40]. Intestinal epithelial cells and kidney proximal tubule cells are notable examples of cells which have these highly specialized brush border microvilli and villin accumulates at their apex. Villin content in differentiated HT29-18 cells, a clone derived from the HT-29 human colonic adenocarcinoma cell line, is 10 times higher than that in undifferentiated HT29-18 cells but close to that seen in normal human colonic cells [41]. Villin is ubiquitously expressed in both crypts and villi of intestinal mucosa. However, during enterocyte differentiation and migration from the crypt to the villous tip, villin translocates from the cytoplasm in the brush border membrane [42]. Thus, although Slfn3 appears to modulate the expression of villin in the jejunal mucosa, expression of villin can be also be driven by environmental factors independent of differentiation. Such a pattern of expression between crypt and villi has been attributed to the cis region of villin [43]. Increased protein level of differentiation marker might reflect either more cells expressing the gene of interest or the same cells express more of the gene of interest. Our in vivo IHC and in vitro work suggests the latter. We did not observe the expansion of the differentiated cells down crypt but individual cells seems to stain more intensively in vivo. Also Caco2 cells in vitro overexpressing Slfn3 seems to express mRNA for Villin and Dpp4.

While the present results suggest that exogenous Slfn3 overexpression or suppression are sufficient to stimulate or reduce intestinal epithelial differentiation, previous preliminary in vitro observations from our lab have suggested that diverse other stimuli also induce differentiation in rat IEC-6 intestinal epithelial cells by increasing Slfn3 [13]. For instance, repetitive deformation of intestinal epithelial monolayers induces an absorptive phenotype in IEC-6 cells cultured in vitro on a type I collagen substrate. This was characterized by increased Dpp4 specific activity and increased villin expression, accompanied by increased Slfn3 expression, and blocked by reducing Slfn3 expression by specific siRNA [13]. Treatment of IEC-6 cells with sodium butyrate or TGF-β similarly seems to increase cellular Dpp4 specific activity in a manner accompanied by and requiring increased Slfn3 expression [13]. It therefore seems plausible that modulation in the regulation of Slfn3 levels by such stimuli may contribute to changes in intestinal epithelial differentiation in vivo in pathologic states such as mucosal atrophy or during enterocytic migration from the crypt to the villus tip. Although in vitro infection of Caco-2 cells with the Slfn3 adenovirus at a high multiplicity of infection induced very high Slfn3 expression, these experiments did validate that the virus actually expressed biologically active Slfn3 in these cells and that the Slfn3 pathway was active in them. The in vivo expression obtained with the virus is more similar to variations in Slfn3 expression obtained in IEC-6 cells in response to more physiologic stimuli such as butyrate, strain and TGF-β.

Changes in Schlafen 3 also accompany intestinal mucosal development in vivo[44]. We demonstrate here that Schlafen 3 is both necessary and sufficient for the expression of several different enterocytic differentiation markers in vivo. Although it is not possible to state that all differentiating agents must act through Schlafen 3 or that all enterocytic differentiation requires Schlafen 3, the wide variety in the differentiating stimuli that appear to induce and require Schlafen 3 and the variety of enterocytic markers that are induced by and require Schlafen 3 all suggest the likely importance of Schlafen 3 as a common denominator in a variety of differentiating pathways. The intracellular mechanisms by which Schlafen 3 induces differentiation are not yet known. Other Schlafen superfamily proteins have been reported to act by inhibiting cyclin D and blocking the cell cycle in G1 (Slfn1)[45], interacting with transfer RNA (SLFN11) [46], regulating invasion and anchorage-independent growth (SLFN5) [47]. Distinguishing among such possibilities awaits further study at the molecular level.

Conversely, although our present and previous results support a substantial role for Slfn3 in the regulation of the rat intestinal epithelial phenotype, there is less evidence that Slfn3 influences intestinal epithelial cell proliferation. We previously found no effect of manipulating Slfn3 on basal or EGF-stimulated IEC-6 cell proliferation [13] and we found no evidence for changes in jejunal epithelial proliferation or apoptosis here in response to Slfn3 overexpression except for a modest increase in villous length. More prolonged Slfn3 overexpression might exert a more readily measurable effect on cell proliferation or apoptosis that could explain the modest increase in villous length, but any such effect would seem less substantial than the effects that we have observed in vivo and in vitro on the intestinal epithelial phenotype.

Villus remodeling in response to starvation [48], dietary changes [49], and other factors [6], [50] has previously been described. The mechanism by which modulation of Schlafen 3 seems to alter villus length awaits elucidation. We have not been able to demonstrate measurable changes in mitotic index or apoptosis (data not shown) with modulation of Schlafen 3 in vitro [13]. However, it is not entirely clear that apoptosis and cell death are the only mechanism by which enterocytes leave the villus. Viable enterocytes may also be sloughed into the lumen off the villus tip before initiation of apoptosis due to disruption of E-cadherin mediated adhesion [51]. Modulation of Schlafen 3 might therefore affect the retention of the enterocyte on the villus, either directly by affecting cell adhesion or indirectly by some juxtacrine mechanism in which the more differentiated enterocyte modulates the function of other cells in the villus. This awaits further study.

Previous work from our laboratory has demonstrated that repetitive deformation can modulate the phenotype of human Caco-2 and rat IEC-6 intestinal epithelial cells [13], [52] by a complex signal pathway that involves p38 [53], [54] and Akt [55], [56] among other intracellular signals. In vitro, the induction of Schlafen 3 by repetitive deformation in rat IEC-6 cells requires both p38 and Akt [13]. Conversely, intestinal defunctionalization would be expected to be accompanied by alterations in patterns of deformation due to the absence of luminal contents. Indeed, this study suggests that such intestinal defunctionalization is also accompanied by decreased p38 and Akt mucosal activation. While this does not prove conclusively that the decreased p38 and Akt signaling causes the decrease in Schlafen 3, this interpretation would be consistent with our present and previous results.

Reduced luminal nutrient flow in the intestine promotes intestinal mucosal atrophy and suppresses mucosal responsiveness to stimuli [17], [57]. Defunctionalization of the intestine reduces expression of the SI and Dpp4 differentiation markers in the mucosa [17]. We used the defunctionalization model of intestinal mucosal atrophy to demonstrate parallel decreases in Glut 2 and villin mucosal differentiation marker expression, Slfn3, and p38 and AKT signaling. We previously reported that deformation-induced Slfn3 expression requires Src-, p38-, and PI3-kinase in vitro, although other stimuli may influence Slfn3 by different signals [13]. However, the downstream cellular pathway by which Slfn3 acts to alter the enterocytic phenotype awaits further definition. Slfn3 lacks a standard nuclear targeting sequence but could bind to a chaperone protein that could bring it into the nucleus, or it could act in some way in the cytosol to influence post-transcriptional mechanisms controlling other key regulators in the determination of intestinal cell fate, such as the caudal-type homeobox transcription factor caudal type homeobox 2 (Cdx2) [58], [59]. Slfn3 may change cyclin-dependent kinase 2 (Cdk-2) expression in HCT-116 cells [60]. Regulation for the Cdx2 transcription factor occurs via phosphorylation of the Cdx2 protein at serine 281 by Cdk-2 and coordinates its polyubiquitination and degradation by the proteasome [59].

In summary, taken together with our previous preliminary in vitro observations, this study demonstrates that Slfn3 is an important regulator of the rat intestinal epithelial phenotype, and may represent a novel common pathway for the regulation of intestinal epithelial differentiation. Further elucidation of how Slfn3 functions may guide the development of new interventions to selectively modulate intestinal epithelial differentiation. These could be used to promote mucosal differentiation, reverse mucosal atrophy and promote intestinal adaptation in patients who have short gut syndromes, either alone or synergistically with mitogenic manipulations such as growth hormone or GLP-2 analogs.
